# Improving outcomes for very preterm babies in England: does place of birth matter? Findings from OPTI-PREM, a national cohort study

**DOI:** 10.1136/archdischild-2024-327474

**Published:** 2024-12-27

**Authors:** Thillagavathie Pillay, Sarah E Seaton, Miaoqing Yang, Vasiliki Bountziouka, Victor Banda, Helen Campbell, Kelvin Dawson, Bradley N Manktelow, Elizabeth S Draper, Neena Modi, Elaine M Boyle, Oliver Rivero-Arias, The OPTI-PREM study group, Matthew Babirecki

**Affiliations:** 1 Faculty of Science and Engineering, University of Wolverhampton, Wolverhampton, UK; 2 Neonatology, University Hospitals of Leicester NHS Trust, Leicester, UK; 3 Department of Population Health Sciences, University of Leicester College of Life Sciences, Leicester, UK; 4 University of Leeds, Leeds, UK; 5 Computer Simulations, Genomics and Data Analysis Laboratory, Department of Food Science and Nutrition, University of the Aegean, Mytilene, Greece; 6 Data, Research, Innovation and Virtual Environment, Great Ormond Street Hospital for Children, London, UK; 7 Nuffield Department of Population Health, University of Oxford National Perinatal Epidemiology Unit, Oxford, UK; 8 Research and Development, BLISS, Royal Wolverhampton Hospitals NHS Trust, Wolverhampton, UK; 9 Neonatal Medicine, School of Public Health, Chelsea and Westminster Hospital Campus, Imperial College London, London, UK

**Keywords:** Mortality, Intensive Care Units, Neonatal, Neonatology

## Abstract

**Objective:**

Babies born between 27^+0^ and 31^+6^ weeks of gestation contribute substantially towards infant mortality and morbidity. In England, their care is delivered in maternity services colocated with highly specialised neonatal intensive care units (NICU) or less specialised local neonatal units (LNU). We investigated whether birth setting offered survival and/or morbidity advantages to inform National Health Service delivery.

**Design:**

Retrospective national cohort study.

**Setting:**

LNU, NICU, England.

**Patients:**

UK National Neonatal Research Database whole population data for births between 27^+0^ and 31^+6^ weeks of gestation, discharged from/died within neonatal units between 1 January 2014 and 31 December 2018. We linked baby-level data to mortality information from the Office for National Statistics.

**Outcome measures:**

Death during neonatal care, up to 1 year (infant mortality), surgically treated necrotising enterocolitis, retinopathy of prematurity, severe brain injury (SBI), bronchopulmonary dysplasia.

**Intervention:**

Birth in NICU versus LNU setting. We used an instrumental variable (maternal excess travel time between the nearest NICU and LNU) estimation approach to determine treatment effect.

**Results:**

Of 18 847 babies (NICU: 10 379; LNU: 8468), 574 died in NICU/LNU care, and 121 postdischarge (infant mortality 3.7%). We found no effect of birth setting on neonatal or infant mortality. Significantly more babies born into LNU settings experienced SBI (mean difference −1.1% (99% CI −2.2% to −0.1%)). This was attenuated after excluding births at 27 weeks, and early postnatal transfers.

**Conclusions:**

In England, LNU teams should use clinical judgement, risk assessing benefits of transfer versus risk of SBI for preterm births at 27 weeks of gestation. 28 weeks of gestation is a safe threshold for preterm birth in either NICU/LNU settings.

**Trial registration number:**

NCT02994849/ISRCTN74230187.

**WHAT IS ALREADY KNOWN ON THIS TOPIC**

- Babies born very preterm (VPT) have a higher risk of mortality and serious morbidity.
- For those born VPT at <27 weeks of gestation, birth in maternity centres colocated with neonatal intensive care unit (NICU) improves outcomes.
- In England, for the next most vulnerable group, born between 27^+0^ and 31^+6^ weeks of gestation, it is unknown whether place of birth and early care influence their outcomes. Here, their birth and early care occur either in maternity services colocated with NICU, or with local neonatal unit (LNU).

**WHAT THIS STUDY ADDS**

- There is no difference in mortality based on place of birth (LNU/non-tertiary vs NICU/tertiary) and early care for VPT babies born between 27^+0^ and 31^+6^ in England.
- For births at 27 weeks of gestation, there is a higher risk of severe brain injury (SBI) when born into maternity services colocated with LNU compared with NICU, and when born into maternity services colocated with low-volume (providing <1614 intensive care days/year) compared with high-volume neonatal units (providing >1614 intensive care days/year). This risk of SBI exists in those transferred out in the first 72 hours after birth.
- Birth and early care for VPT babies can safely be provided closer to home in maternity centres colocated with LNU and NICU from 28 weeks onwards.

**HOW THIS STUDY MIGHT AFFECT RESEARCH, PRACTICE OR POLICY**

- To reduce risks associated with SBI in VPT, anticipated preterm births at 27 weeks of gestation should be targeted for delivery in maternity centres colocated with NICU in England.
- Specific consideration should be given to anticipated preterm births at 27 weeks of gestation for birth in maternity centres colocated with neonatal units providing a high volume of intensive care days.
- Study outcomes may guide UK policy and practice, contributing to more effective redistribution of birth and care of VPT babies, and the development of capacity within NICU in the UK.

## Introduction

 Babies born very preterm (VPT, <32 weeks of gestation) require skilled care in the first few weeks of life, and are at greatest risk of morbidity and mortality.^1^,^3^ For extreme preterm births at <27 weeks of gestation in the UK, outcomes are significantly improved if born into maternity services colocated with highly specialised neonatal intensive care units (NICU).^4^ For the next most vulnerable group born between 27^+0^ and 31^+6^ weeks of gestation, optimal place of birth remains uncertain.

VPT birth is distributed almost equally between maternity services colocated with the highly specialised/tertiary NICU, or into less specialised/non-tertiary local neonatal units (LNU).^5^ NICUs are generally resourced to provide tertiary-level care for sick babies across all gestational ages and across all complexities of care. LNUs provide care for neonates in their local catchment area; these are not configured to provide long-term intensive care but can provide emergency and short-term intensive support for sick babies, and up to the present, can care for VPT babies generally born at >27 weeks of gestation. Between 2014 and 2018, there were 82 LNUs and 43 NICUs in England. For VPT born between 27^+0^ and 31^+6^ weeks of gestation, place of birth is dependent on mother’s choice of maternity service at booking, presentation to the nearest hospital, anticipated maternal/fetal/neonatal complications and availability of resources for perinatal care. In England, 4.7% of babies in this age group (born at 27–31 weeks of gestation) do not survive after admission to neonatal services^6^; those who do spend a median of 34–79 days in neonatal units.^6^ In 2016, they accounted for 5988 admissions at an estimated National Health Service (NHS) cost of £262 million per annum.^7^

With increasing demand for capacity in neonatal services,^8 9^ there is an urgent need to identify whether there is an optimal place of birth for this group of babies. We report on the National Institute for Health and Care Research (NIHR)-funded OPTI-PREM, evaluating optimal place of birth for VPT babies born between 27^+0^ and 31^+6^ weeks of gestation in England.^10^

## Methods

### Study design and participants

The OPTI-PREM cohort (NCT02994849/ISRCTN74230187) comprised all babies born between 27^+0^ and 31^+6^ weeks of gestation in maternity services colocated with the 43 NICUs or 81 LNUs in England, who were discharged from or died in neonatal units between 1 January 2014 and 31 December 2018. Neonatal unit admissions were identified from the National Neonatal Research Database (NNRD) which holds data on babies admitted to NHS neonatal units in England, Wales, Scotland and the Isle of Man. Mortality data up to 1 year of age were identified through linkage to the Office for National Statistics.

We excluded babies with any of the following: (a) a major congenital anomaly (online supplemental material 1), (b) missing data for maternal and/or baby characteristics, (c) born outside of a maternity service colocated with LNU or NICU, (d) admitted to one LNU that declined participation in OPTI-PREM and (e) 10 parents from across the country who declined inclusion of their baby’s data in the OPTI-PREM study. The study was publicised through unit posters and leaflets. Opt-out consent was obtained.

### Neonatal and infant outcomes

Primary outcomes were death in neonatal care and in the first year (infant mortality). Secondary outcomes were retinopathy of prematurity (ROP), severe brain injury (SBI), surgically treated necrotising enterocolitis (NEC), bronchopulmonary dysplasia (BPD) and one measure of clinical care (receipt of any human breast milk feeds (BMF) at discharge from neonatal care). A composite outcome comprising any of ROP, SBI, NEC, BPD and death was also assessed. Online supplemental material 2 provides definitions for all outcomes.

### Statistical methods

In the absence of randomised allocation of babies to each neonatal unit designation (ie, LNU, NICU) alternative statistical methods were required to address biases likely arising from a non-randomised comparison of outcomes of babies receiving care in each setting.

### Preliminary propensity score matching

We initially employed a propensity score matching approach to create a sample of babies born in each setting, balanced in terms of neonatal and maternal characteristics. The matching exercise (online supplemental material 3) lacked validity due to limited data on confounders. Unmeasured characteristics that varied by unit designation could have further biased estimates of outcomes, if left unaddressed. Therefore, an instrumental variable (IV) approach was employed.^11^

### IV approach

Our IV approach aimed to identify a variable that mimics women delivering in a particular setting randomly, thereby controlling naturally for both measured and unmeasured confounders. This variable is referred to as an instrument and in line with previous epidemiological studies of observational data, we considered travel time to a facility, specifically the difference in the travel time between the mother’s residential postcode and her nearest NICU postcode, and the mother’s residential postcode and her nearest LNU postcode (known henceforth as the excess travel time to a NICU).^12^

To compare outcomes by unit designation, IV models were estimated for each outcome in Stata MP V.18 using a bivariate probit regression.^13^ Outcomes and ‘unit designation’ (NICU or LNU) were included as binary variables and excess travel times as a continuous variable. The models included the measured confounders used in the propensity score matching exercise (online supplemental material 3). A 1% statistical significance level (p≤0.01) was selected with 99% CIs presented accordingly. Extended methods are presented in online supplemental materials 4 and 5.

### Sensitivity analyses

Three separate sensitivity analyses were conducted.^10^ The first repeated the main analyses after excluding babies transferred out in the first 72 hours (early transfers). This helped us understand whether our results were disproportionately influenced by babies born in LNU settings, whose condition was severe enough requiring transfer of care to NICU. Babies who were not transferred and had not died within this 72-hour time frame were treated as representative of those receiving early care in their respective units.

A second sensitivity analysis excluded babies born to mothers with multiple pregnancies; the rationale being that maternity services colocated with a NICU are more likely to deal with complex multiple pregnancies that carry a higher risk of neonatal mortality/morbidity.

To avoid overlooking higher performing LNU working on par with NICU and vice versa, a third analysis compared outcomes for babies born and cared for in high-volume units with those cared for in low-volume neonatal units.^14^ High-volume units were identified as those above the upper quartile for number of intensive care bed days offered to VPT babies during the OPTI-PREM study period (those providing >1614 intensive care days/year). Units below the upper quartile were defined as low-volume units.^14^

### Parent involvement

OPTI-PREM established a parent panel through Bliss, the national charity for babies born premature or sick.^15^ This was an ethnically diverse group of 10 parents (mothers or fathers) of babies born between 27^+0^ and 31^+6^ weeks of gestation in England, including those who had experienced neonatal death, and/or transfers between neonatal units. The panel engaged in study design, leaflet development, team and study committee meetings, and stakeholder discussions, and are active participants in the dissemination phase of OPTI-PREM.

## Results

**Figure 1:**
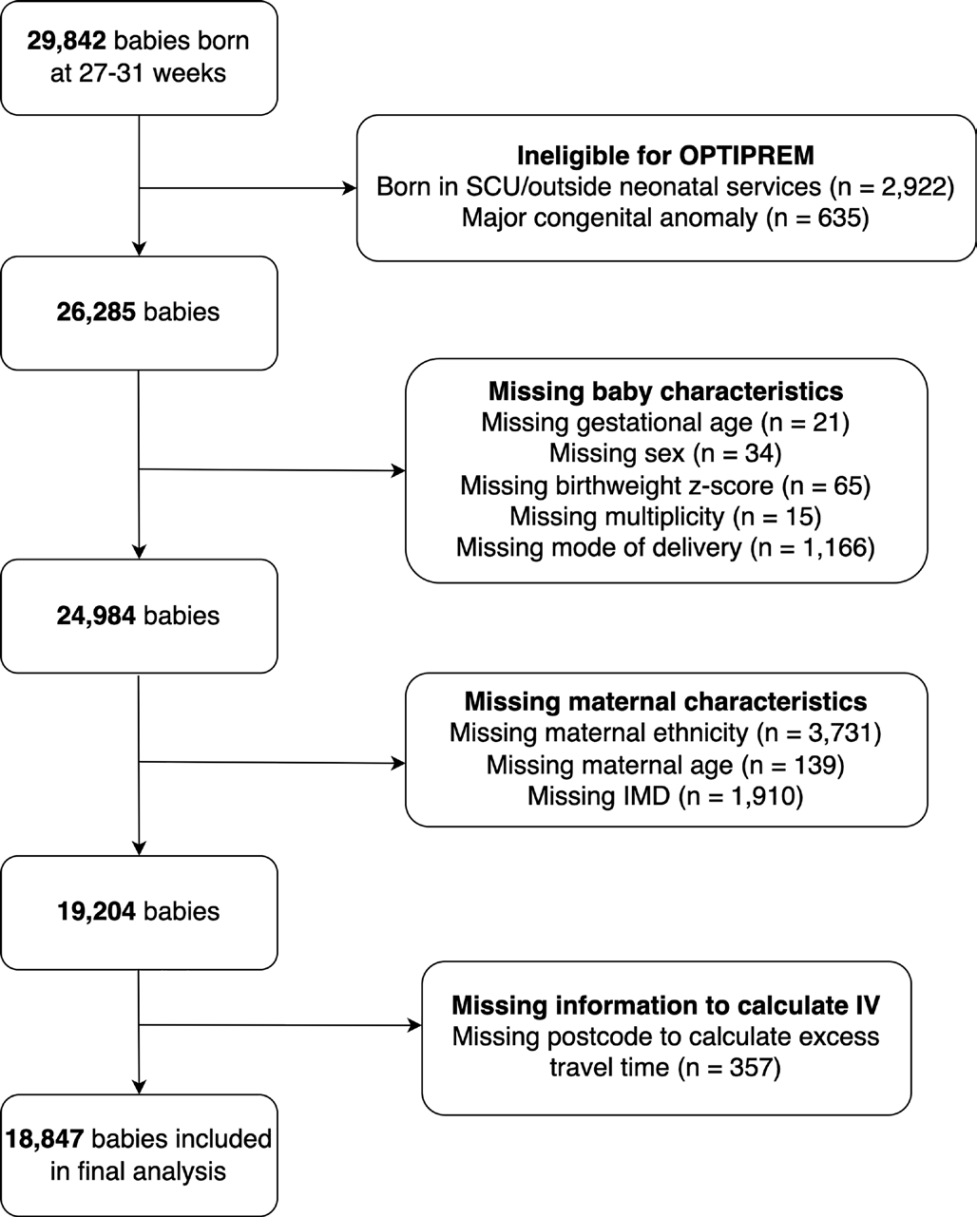
OPTI-PREM data study flow chart. IMD, Index of Multiple Deprivation; IV, instrumental variable; SCU, special care baby unit.

From the NNRD data extraction on 29 842 babies, 18 847 were included in the analysis; 10 379 were born in maternity services colocated with NICU and 8468 to LNU (figure 1). A comparison of this cohort with babies excluded due to missing data (n=7438) is described in online supplemental material 6.

Table 1 shows a greater proportion of babies born at 27 weeks of gestation into NICU settings, with LNU settings receiving higher proportions of babies born at later gestations. Babies admitted into NICU were lighter in weight and more likely to have been part of a multiple pregnancy than babies admitted to LNU. Table 1 shows mothers who delivered their preterm babies in maternity services colocated with NICU were more likely to be of mixed or ethnic minority groups and had higher levels of social deprivation and higher caesarean section rates.

**Table 1:** Neonatal and maternal characteristics by place of birth (maternity unit colocated with a neonatal intensive care unit (NICU) or a local neonatal unit (LNU))

	NICU (n=10 379)	LNU (n=8468)
**Neonatal characteristics**		
Gestational age, n (%)		
27 weeks	1507 (14.5)	777 (9.2)
28 weeks	1773 (17.1)	1258 (14.9)
29 weeks	1885 (18.2)	1527 (18.0)
30 weeks	2348 (22.6)	2031 (24.0)
31 weeks	2866 (27.6)	2875 (34.0)
Birth weight (g)	1286 (342.5)	1356 (312.4)
Male sex, n (%)	5658 (54.5)	4659 (55.0)
Singleton, n (%)	7254 (69.9)	6318 (74.6)
Temperature on admission	36.9 (0.6)	36.8 (0.6)
Missing*	100	70
Apgar score at 5 min	9 (6 to 10)	9 (6 to 10)
Missing	667	557
Antenatal steroids provided, n (%)	9538 (92.7)	7737 (92.0)
Missing	98	62
Died in neonatal care, n (%)	391 (3.8)	183 (2.2)
27 weeks	120 (8.0)	48 (6.2)
28 weeks	115 (6.5)	57 (4.5)
29 weeks	53 (2.8)	36 (2.4)
30 weeks	41 (1.8)	23 (1.1)
31 weeks	62 (2.2)	19 (0.7)
Died in 1 year, n (%)	468 (4.5)	227 (2.7)
27 weeks	141 (9.4)	53 (6.8)
28 weeks	127 (7.2)	66 (5.3)
29 weeks	70 (3.7)	42 (2.8)
30 weeks	52 (2.2)	34 (1.7)
31 weeks	78 (2.7)	32 (1.1)
Died in neonatal care (singleton births)	300 (4.1)	156 (2.5)
Died in neonatal care (multiple births)	91 (2.9)	27 (1.3)
Died in 1 year (singleton births)	353 (4.9)	183 (2.9)
Died in 1 year (multiple births)	115 (3.7)	44 (2.1)
**Maternal characteristics**		
Caesarean section, n (%)	7132 (68.7)	5625 (66.4)
Maternal ethnicity, n (%)		
White	7457 (71.8)	6338 (74.8)
Black	956 (9.2)	712 (8.4)
Asian	1532 (14.8)	1106 (13.1)
Mixed	210 (2.0)	149 (1.8)
Other	224 (2.2)	163 (1.9)
Maternal age	31 (6.3)	31 (6.2)
Maternal IMD decile, n (%)		
1 (least deprived)	1304 (12.6)	1210 (14.3)
2	1447 (13.9)	1318 (15.6)
3	1630 (15.7)	1677 (19.8)
4	2246 (21.6)	2086 (24.6)
5 (most deprived)	3752 (36.2)	2177 (25.7)

Data are shown as mean (SD) or median (10th, 90th centiles) unless otherwise indicated.

* Any temperature <33°C or >39°C was regarded as missing.

IMD, Index of Multiple Deprivation.

With imbalances observed between settings in maternal and baby characteristics, we categorised the cohort instead using the study’s IV (median 3.91 (range −130.36 to 74.11) min). The median excess travel time to a NICU (3.9 min) was used as the cut-off value, with travel times <3.9 min suggesting birth in a NICU setting and travel time >3.9 min birth in an LNU setting. Table 2 shows the groups now appeared well balanced in all categories except for the Index of Multiple Deprivation (IMD) scores. Women giving birth in maternity services colocated with NICU were more likely to have greater levels of deprivation than those in an LNU setting (standardised difference=0.305).

**Table 2:** Neonatal and maternal characteristics by median excess travel time to a neonatal intensive care unit (NICU)

	Excess travel time*		Standardised difference†
	<3.9 min (n=9420)	≥3.9 min (n=9427)	
**Neonatal characteristics**			
Gestational age, n (%)			
27 weeks	1115 (11.8)	1169 (12.4)	0.020
28 weeks	1505 (16.0)	1523 (16.2)	
29 weeks	1718 (18.2)	1694 (18.0)	
30 weeks	2210 (23.5)	2169 (23.0)	
31 weeks	2872 (30.1)	2869 (30.4)	
Birth weight (g)	1320 (332)	1314 (330)	0.016
Male sex, n (%)	5146 (54.6)	5171 (54.9)	0.005
Singleton, n (%)	6821 (72.4)	6751 (71.6)	0.018
Temperature on admission	36.9 (0.6)	36.8 (0.6)	0.102
Missing*	87	83	
Apgar score at 5 min	9 (6 to 10)	9 (6 to 10)	0.021
Missing	617	607	
Antenatal steroids provided, n (%)	8577 (91.9)	8698 (93.0)	0.041
Missing	87	73	
Died in neonatal care, n (%)	288 (3.1)	286 (3.0)	0.001
27 weeks	80 (7.2)	88 (7.5)	0.014
28 weeks	83 (5.5)	89 (5.8)	0.014
29 weeks	43 (2.5)	46 (2.7)	0.013
30 weeks	35 (1.6)	29 (1.3)	0.021
31 weeks	47 (1.6)	34 (1.2)	0.038
Died in 1 year, n (%)	346 (3.7)	349 (3.7)	0.002
27 weeks	95 (8.5)	99 (8.5)	0.002
28 weeks	93 (6.2)	100 (6.6)	0.015
29 weeks	53 (3.1)	59 (3.5)	0.022
30 weeks	45 (2.0)	41 (1.9)	0.011
31 weeks	60 (2.1)	50 (1.7)	0.025
Died in neonatal unit (singleton births)	227 (3.3)	229 (3.4)	0.004
Died in neonatal unit (multiple births)	61 (2.4)	57 (2.1)	0.015
Died in 1 year (singleton births)	269 (3.9)	267 (4.0)	0.001
Died in 1 year (multiple births)	77 (3.0)	82 (3.1)	0.006
**Maternal characteristics**			
Caesarean section, n (%)	6304 (66.9)	6453 (68.5)	0.033
Maternal ethnicity, n (%)			
White	6827 (72.5)	6968 (73.9)	0.070
Black	794 (8.4)	874 (9.3)	
Asian	1420 (15.1)	1218 (12.9)	
Mixed	192 (2.0)	167 (1.8)	
Other	187 (2.0)	200 (2.1)	
Maternal age	30.7 (6.2)	31.0(6.2)	−0.060
Maternal IMD decile, n (%)			
1 (least deprived)	1091 (11.6)	1423 (15.1)	0.305
2	1242 (13.2)	1523 (16.2)	
3	1439 (15.3)	1868 (19.8)	
4	2033 (21.6)	2299 (24.4)	
5 (most deprived)	3615 (38.4)	2314 (24.5)	

Data are shown as mean (SD) or median (10th, 90th centiles) unless otherwise indicated.

* Instrument used for this study representing additional travel time women would need to travel beyond the nearest local neonatal unit (LNU) to arrive at a hospital with NICU. Median excess travel time was 3.9 min.

† Absolute standardised difference of ≥0.10 generally indicates that covariates are imbalanced between groups.

IMD, Index of Multiple Deprivation.

### Mortality

There were 574 deaths (3.0%) in NICU and LNU care, and a further 121 deaths in the first year following discharge (total mortality 3.7%). Babies admitted to NICU had a higher unadjusted mortality while receiving neonatal care (3.8% for NICU vs 2.2% for LNU; p<0.001) and higher unadjusted infant mortality (4.5% for NICU and 2.7% for LNU; p<0.001). After adjustment using IV modelling, the mean difference in mortality for babies born into NICU versus LNU settings was −0.1% (99% CI −1.1% to 1.0%) while receiving neonatal care, and −0.2% (99% CI −1.4% to 0.9%) for overall infant mortality (table 3). There was no mortality difference by gestational week at birth.

**Table 3:** Association between place of birth (maternity unit colocated with a neonatal intensive care unit (NICU) or a local neonatal unit (LNU)) and overall and gestational age-specific mortality risk while in neonatal care and at 1 year using instrumental variable model

	Case (n)	Sample size	NICU mean percentage (SE)	LNU mean percentage (SE)	Adjusted mean percentage difference (99% CI)*	P value for difference
Died in neonatal unit						
Overall	574	18 847	3.0% (0.2%)	3.1% (0.3%)	−0.1% (−1.1% to 1.0%)	0.8
27 weeks	168	2284	6.9% (0.8%)	8.6% (0.2%)	−1.7% (−8.1% to 4.8%)	0.5
28 weeks	172	3031	5.2% (0.6%)	6.8% (1.3%)	−1.6% (−5.6% to 2.4%)	0.3
29 weeks	89	3412	2.3% (0.4%)	3.2% (0.7%)	−0.9% (−3.1% to 1.4%)	0.3
30 weeks	64	4379	1.4% (0.3%)	1.6% (0.4%)	−0.2% (−1.7% to 1.3%)	0.7
31 weeks	81	5741	1.8% (0.3%)	0.9% (0.2%)	0.9% (−0.2% to 1.9%)	0.03
Died in 1 year						
Overall	695	18 847	3.6% (0.2%)	3.8% (0.3%)	−0.2% (−1.4% to 0.9%)	0.6
27 weeks	194	2284	8.3% (0.9%)	8.9% (2.0%)	−0.5% (−7.1% to 6.0%)	0.8
28 weeks	193	3031	5.7% (0.6%)	7.8% (1.3%)	−2.1% (−6.2% to 2.1%)	0.2
29 weeks	112	3412	2.9% (0.4%)	4.0% (0.8%)	−1.0% (−3.5% to 1.4%)	0.3
30 weeks	86	4379	1.8% (0.3%)	2.3% (0.5%)	−0.5% (−2.3% to 1.3%)	0.5
31 weeks	110	5741	2.2% (0.3%)	1.5% (0.3%)	0.7% (−0.6% to 1.9%)	0.2

* Adjusted for gestational age (when analysing the overall cohort), sex, birth weight z-score, multiplicity, mode of delivery, maternal ethnicity, maternal age and Index of Multiple Deprivation.

In total, 6016 (31.9%) babies were transferred to another unit for ongoing care; 1545 within the first 72 hours of life. A descriptive analysis of transfers, including direction of transfers in the first 72 hours, is provided in online supplemental material 7. Of 1545 transfers in the first 72 hours, 928 (60.1%) were transferred out of LNU; of these, 834 (89.9%) were transfers from LNU to NICU. There were a total of 2284 births at 27 weeks of gestation, of whom 310 (13.5%) were transferred in the first 72 hours of birth. 228 of these (73.5%) were transfers out of LNU, of which 219 (96.1%) were uplifts to NICU (LNU to NICU). There were 26 capacity transfers (17 NICU to LNU transfers, 9 LNU to LNU transfers).

Sensitivity analyses conducted after excluding these early transfers found no significant differences between NICU and LNU settings in mortality while in neonatal care (adjusted mean difference 0.2%; 99% CI −0.9% to 1.3%) and up to 1 year (adjusted mean difference 0.0%; 99% CI −1.2% to 1.2%).

All high-volume units were NICU. Analyses comparing high-volume and low-volume units (independent of NICU and LNU designation) found no significant differences in mortality (online supplemental material 8). No mortality differences were found in the analysis of singleton births. Transfers for high-volume versus low-volume units for each gestational age are detailed in online supplemental material 7—table 2.

### Secondary outcomes

Adjusted analyses of the secondary outcomes are displayed in table 4, with additional information in online supplemental material 9. Place of birth had no impact on ROP, NEC, BMF or composite outcomes. This finding held when analyses were performed by individual gestational week at birth, and on excluding early transfers and multiple births.

**Table 4:** Risks associated with place of birth (maternity unit colocated with a neonatal intensive care unit (NICU) or a local neonatal unit (LNU)) and key secondary outcomes using instrumental variable model

	Case (n)	Sample size	NICU mean percentage (SE)	LNU mean percentage (SE)	Adjusted mean percentage (99% CI)*	P value for difference
Any morbidity† or died	3407	18 847	18.4% (0.4%)	17.8% (0.6%)	0.6% (−1.6% to 2.8%)	0.5
ROP	297	17 930	1.7% (0.2%)	1.6% (0.2%)	0.2% (−0.7% to 1.0%)	0.6
BPD	1819	18 273	10.7% (0.4%)	8.9% (0.4%)	1.8% (0.1% to 3.5%)	0.006
NEC	490	18 847	2.6% (0.2%)	2.6% (0.3%)	0.0% (−1.1% to 1.1%)	0.96
SBI	735	18 847	3.4% (0.2%)	4.5% (0.3%)	−1.1% (−2.2% to −0.1%)	0.007
BMF	10 220	18 273	55.9% (0.6%)	55.8% (0.7%)	0.1% (−2.8% to 3.1%)	0.9
Excluding babies born at 27 weeks						
Any morbidity† or died	2344	16 563	14.3% (0.4%)	14.0% (0.5%)	0.3% (−1.8% to 2.4%)	0.7
ROP	174	15 891	1.1% (0.2%)	1.1% (0.2%)	0.1% (−0.7% to 0.8%)	0.8
BPD	1137	16 157	7.5% (0.3%)	6.4% (0.4%)	1.1% (−0.4% to 2.7%)	0.06
NEC	363	16 563	2.1% (0.2%)	2.3% (0.3%)	−0.2% (−1.2% to 0.9%)	0.7
SBI	529	16 563	2.8% (0.2%)	3.7% (0.3%)	−0.8% (−1.9% to 0.2%)	0.04
BMF	9185	16 157	57.0% (0.7%)	56.4% (0.7%)	0.5% (−2.5% to 3.6%)	0.7

Babies may have more than one morbidity and so may appear as a case in multiple analyses.

* Adjusted for gestational age, sex, birth weight z-score, multiplicity, mode of delivery, maternal ethnicity, maternal age and Index of Multiple Deprivation.

† ROP or BPD or SBI or NEC.

BMF, breast milk feeds at time of discharge from neonatal unit; BPD, bronchopulmonary dysplasia; NEC, surgically treated necrotising enterocolitis; ROP, retinopathy of prematurity; SBI, serious brain injury.

A significantly higher proportion of SBI was identified in babies born in LNU settings (adjusted mean difference −1.1%; 99% CI −2.2% to −0.1%; table 4). This significance was lost on exclusion of babies transferred to other units within 72 hours of birth.

Babies born at the earliest gestations were at higher risk of an early transfer and SBI (online supplemental material 7); 310 of 2284 babies born at 27 weeks of gestation (13.6%) underwent an early transfer, of whom 45 (14.5%) had SBI. In contrast, 368 of 5741 babies born at 31 weeks of gestation (6.4%) underwent early transfer, of whom 13 (3.5%) had SBI. In an adjusted comparative analysis of SBI conducted after excluding babies born at 27 weeks of gestation, the difference in SBI previously seen was no longer significant (mean difference −0.8%; 99% CI −1.9% to 0.2%; table 4). For babies born at 27 weeks of gestation, birth in a NICU reduced the risk of SBI from 11.9% to 8.0%, a statistically non-significant adjusted mean difference of 4.0% (99% CI −9.6% to 1.7%).

42 (93.3%) of 45 babies born at 27 weeks of gestation with early transfer and SBI were transferred out of low-volume units. For babies born at 27 weeks of gestation, birth in a high-volume unit significantly reduced the risk of SBI from 24.2% to 2.8% (adjusted mean difference 28.9%; 99% CI 3.5% to 54.2%; online supplemental material 10).

The rate of BPD was higher in those born into NICU settings (adjusted mean difference 1.8%; 99% CI 0.1% to 3.5%; table 4). This difference remained significant after excluding early transfers up to 72 hours after delivery. The difference in BPD was greatest in babies born at 27 weeks of gestation. When this gestational age group was excluded from the analyses, the significant difference was lost (adjusted mean difference 1.1%; 99% CI −0.4% to 2.7%; table 4).

## Discussion

OPTI-PREM found no significant differences in mortality outcomes for births between 27*^+^*^0^ and 31^+6^ weeks of gestation in NICU or LNU settings. However, location of birth was important for morbidity. There was an increased risk for SBI for babies born at 27 weeks of gestation in LNU settings. There was also a significant association between SBI and early postnatal transfer out of LNU. These findings indicate that births at 27 weeks of gestation should occur in maternity services colocated with NICU (preferably those with a high volume of intensive care days/year), and that a reasonable threshold for VPT births in maternity services closer to home, regardless of whether it is colocated with a NICU or LNU, is 28 weeks of gestation.

The increased risk of SBI in babies born at 27 weeks of gestation in LNU settings is of concern. With associated adverse outcomes of SBI, the NHS and lifetime societal costs are substantial.^316^,^19^ Managed clinical networks have facilitated the provision of perinatal care closer to home,^20^,^24^ but brought with it increased postnatal transfer of unexpectedly sick preterm babies between neonatal units. OPTI-PREM confirms a high rate of postnatal transfers in babies born between 27^+0^ and 31^+6^ weeks of gestation and demonstrates the negative effect of this, especially in those born at 27 weeks of gestation. This adds to global evidence on risks of postnatal transfer of VPT babies.^25^,^27^ We were unable to identify the specific timing of the SBI in relation to transfers out of LNU. These could be related to the process of transfer, differences in clinical profile of babies born at 27 weeks of gestation at maternity centres colocated with LNU, or clinical experience or care provided at LNU which see fewer babies born at 27 weeks of gestation than NICU.

There will be situations where the birth of a high-risk VPT baby in LNU settings is unavoidable, for example, if maternal illness or presenting stage of labour precludes transfer. OPTI-PREM findings of an association between early postnatal transfer and SBI at 27 weeks of gestation suggest that local neonatal teams should use clinical judgement, risk assessing the benefit of transfer versus risk of SBI.

Our findings raise challenges for perinatal teams. It is not always possible a priori to predict which babies born at 27 weeks of gestation in LNU will require postnatal transfer to NICU, and which of these are at greatest risk of developing SBI. The logical recommendation would be to promote antenatal transfer of all anticipated preterm births at 27 weeks of gestation to maternity services colocated with a NICU; however, adequate capacity for expectant mothers at these centres is a major issue in the UK.^28^ Therefore, for preterm births at 27 weeks of gestation in LNU, clinical teams should use careful clinical judgement, risk assessing the benefit of transfer versus the risk of SBI. The health economics implications are also important and will be evaluated separately.

OPTI-PREM’s strengths include utility of national quality assured operational data and statistical methodologies that facilitated comparison between unit designations, adjusting for measured and unmeasured confounders.^29^ Further strengths include extensive sensitivity analyses conducted around transfers and multiple births and the inclusion of infant mortality at 1 year.

Its limitations include missing data, mainly relating to mode of delivery, maternal ethnicity and IMD. Incomplete data submitted to the NNRD meant we were unable to incorporate observable confounder information on major maternal comorbidities (pregnancy-induced hypertension, diabetes, chorioamnionitis and twin-to-twin transfusion). Therefore, our analysis is subject to residual confounding due to missing information. However, the well-balanced distribution of measured confounders achieved by our instrument led us to believe that similar distribution may have been achieved for unobservable confounders. We were unable to study deaths in the delivery suite because of incomplete entries in neonatal electronic records. For clinical morbidities, where there were no entries, these were assumed as not present as it was not possible to evaluate whether they were truly missing. A further limitation is that only core neonatal outcomes were selected for study. Length of stay and other National Neonatal Audit Programme measures were investigated as part of the OPTI-PREM programme of work, and will be reported elsewhere (NIHR draft report, in press). We also acknowledge the usual concerns about the use of IV estimation to determine causal effects,^30^ including the impact of using weak instruments on model coefficients and the assumptions needed to operationalise the framework. The extended methods presented in online supplemental material 4 provide evidence to support our choice of instrument and the validity of these assumptions.

The statistical significance for BPD for births in NICU requires further study including evaluation of ventilation strategies, familiarity with optimal early care for the preterm baby. Since BPD is a known risk factor for later adverse neurodevelopment outcome, it will also be important to balance the increased risk of SBI in LNU settings with increased risk of BPD in NICU settings through further research in this area.

## Conclusions

OPTI-PREM identified 28 weeks of gestation as a safe threshold for preterm birth within either LNU or NICU settings. For VPT at 27 weeks of gestation, we found a higher risk of SBI with early postnatal transfers (within 72 hours of birth) out of LNU at 27 weeks, with no difference in outcomes for births between 28^+0^ and 31^+6^ weeks of gestation in LNU versus NICU. As neonatal services deal with increasing demand and capacity pressures, OPTI-PREM provides objective information towards optimising neonatal service delivery in England.

## Supplementary material

10.1136/archdischild-2024-327474 online supplemental file 1

## Data Availability

All data relevant to the study are included in the article or uploaded as supplementary information.
