# Supplementary material for: Improving outcomes for very preterm babies in England: does place of birth matter? Findings from OPTI-PREM, a national cohort study
Source: Arch Dis Child Fetal Neonatal Ed. 2024 Dec 27;110(5):e327474. doi: 10.1136/archdischild-2024-327474 (PMC12418565; doi:10.1136/archdischild-2024-327474)
Supplement: online supplemental file 1 [file fetalneonatal-110-5-s001.pdf]

Improving outcomes for very preterm babies in England: does place of birth matter? Findings from OPTI-PREM, a national cohort study

Supplementary material

Table of contents

Supplementary material 1: List of major congenital anomalies 2

Supplementary material 2: Definition of secondary outcomes studied 4

Supplementary material 3: Preliminary propensity score matching 5

Supplementary material 4: The instrumental variable modelling framework - key considerations and interpretation of model outputs 10

Supplementary material 5: Estimation of differential travel time (excess travel time) to a neonatal intensive care unit (NICU) 15

Supplementary material 6: Comparison of cohort excluded due to missing data with those included in the final analysis 16

Supplementary material 7: Additional information about transfers within 72 hours of life 17

Supplementary materials 8: Association between place of birth (high volume admissions versus low volume admissions) and overall and gestational age-specific mortality risk whilst in neonatal care and at one year using instrumental variable model (n=18,781) 22

Supplementary material 9: Distribution of actual cases for the secondary outcomes in preterm babies born at 27-31 weeks gestation in maternity services co-located with NICU and LNU, and admitted into neonatal care 23

Supplementary material 10: Association between place of birth (high volume admissions versus low volume admissions) of risk of serious brain injury whilst in neonatal care under different scenarios using instrumental variable model 24

Supplementary material 1: List of major congenital anomalies

Atresia and stenosis of small intestine
Atresia of bile ducts
Atresia of oesophagus with tracheo-oesophageal fistula
Atresia of oesophagus without fistula
Atresia of urethra
Atrioventricular septal defect (AVSD)
Coarctation of aorta
Coarctation of the aorta
Congenital absence, atresia, and stricture of auditory canal (external)
Congenital absence, atresia/stenosis of anus with/without fistula
Congenital absence, atresia/stenosis of rectum with/without fistula
Congenital cardiac disease - acyanotic
Congenital cardiac disease - non-cyanotic
Congenital malformations of aortic and mitral valves
Congenital malformations of cardiac chambers and connections
Congenital malformations of pulmonary and tricuspid valves
Down syndrome (translocation)
Down syndrome (Trisomy 21)
Down's syndrome
Edwards syndrome (Trisomy 18)
Encephalocele
Encephalocele (unknown or unspecified cause)
Eventration of diaphragmatic hernia
Eventration of the diaphragm
Exomphalos
Exomphalos (major)
Exomphalos (minor)
Exomphalos Malrotation
Exotrophy of urinary bladder
Gastroschisis
Hypoplasia of aortic arch
Malformation of aorta
Oesophageal atresia
Oesophageal atresia with distal tracheal fistula
Oesophageal atresia with tracheoesophageal fistula
Oesophageal atresia without distal fistula
Other congenital malformations of aortic arch
Polycystic kidney
Polycystic kidney (unknown or unspecified cause)

Potter's syndrome
Spina bifida
Spina bifida (unspecified)
Stenosis of aorta (AS)
Stenosis of pulmonary artery (PS)
Tetralogy of Fallot
Total anomalous pulmonary venous connection
Total anomalous pulmonary venous drainage
Transposition great arteries (TGA)
Trisomy 18
Trisomy 21

Supplementary material 2: Definition of secondary outcomes studied

Outcome	Definition
ROP	A baby with Stage 3+ retinopathy of prematurity in one or both eyes (severe ROP) while within neonatal care. All babies who had laser surgery were also included on the assumption that they would have Stage 4 ROP to warrant surgery.
SBI	A baby having one or more of the following at any time while in neonatal care: periventricular leukomalacia, unilateral or bilateral grade 3 or 4 intraventricular haemorrhage, porencephalic cysts or hydrocephalus. The diagnosis of SBI is usually made on cranial ultrasonography.
NEC	Surgically treated necrotising enterocolitis: A baby undergoing surgical treatment/laparotomy for NEC while in neonatal care, with information extracted from the abdominal X-ray table in the NNRD data and supplemented with information reviewed in the entries on daily data of necrotising enterocolitis. Cases where suspected necrotising enterocolitis was recorded were excluded.
BPD	Oxygen dependence at 36 weeks postmenstrual age, and a requirement for oxygen for at least 80% of the days before reaching 36 weeks post menstrual age. Babies who were discharged before 36 weeks post conceptional age were regarded as not oxygen dependent. In England, babies who were oxygen dependent would not be discharged before 36 weeks. The data were extracted using the added oxygen variable within the daily data.
BMF	Any human breast milk feed administered to baby on the last day of neonatal admission. This was extracted from the final day records on the NNRD.
Place of care for babies	A baby's care was assigned to NICU or LNU according to whether the maternity service in which they were born was co-located with NICU or LNU.
Composite outcome	Outcome indicator comprising any of ROP, SBI, NEC, BPD and death.

BPD, bronchopulmonary dysplasia; BMF, breast milk feeds at time of discharge from neonatal unit; NEC, necrotising enterocolitis; ROP, retinopathy of prematurity; SBI, serious brain injury.

Supplementary material 3: Preliminary propensity score matching

Methods

Our starting point for the statistical analysis was to undertake a propensity score matching to account for measured confounders to explain differences between babies born in NICU (treated units) and babies born to LNU (untreated units) and to estimate average treatment effects for the treated (ATET).[1,2] The ATET measures the difference in mean (average) outcomes between participants assigned to the treated units (i.e. NICU) and units assigned to the untreated units (i.e. LNU). Propensity score matching was implemented using the nearest neighbour algorithm with no caliper and replacement in the LNU group. Using replacement allowed single cases in the untreated units to be matched to multiple treated units to facilitate the matching process. We matched babies born in NICU (treated) and babies born in LNU (untreated) based on variables selected a priori by our Study Steering Committee. These variables were: sex (male/female); gestational age (grouped as 27^{+0} to 27^{+6} weeks; 28^{+0} to 28^{+6} weeks; 29^{+0} to 29^{+6} weeks; 30^{+0} to 30^{+6} weeks and 31^{+0} to 31^{+6} weeks); birthweight z-score (calculated using updated birthweight centiles for England and Wales)[3], multiplicity (singleton versus multiple birth), mode of delivery (grouped as: caesarean versus vaginal delivery), mother's age at birth, mother's ethnic background (grouped as: White/Black/Asian/Mixed/Other) and Index of Multiple Deprivation (IMD: grouped into quintiles from most to least deprived).

To identify matches, the Euclidean distance based on the continuous variable's birthweight and maternal age at birth was used allowing for a difference of 0.5 standard deviations between matched individuals. For these variables, we used a bias-correction term, as recommended in the literature.[4] Exact matching was used for the categorical variables to force 'treated' and 'untreated' to have the same sex, gestational age, multiplicity, mode of delivery, maternal ethnicity and IMD quintile. Robust standard errors for the ATET were calculated using the independent and identically distributed assumption, which ensures that the outcome and treatment status of each individual are unrelated to the outcome and treatment status of all the other individuals in the population.[4] Successful matching was ensured by checking the standardised differences and variance ratios of the matching variables between the two groups.

Sensitivity analyses

An additional important variable selected for inclusion in the matching exercise by the Study Steering Committee was the presence of maternal medical conditions. There were four key medical conditions that we considered important to include in the analysis: Maternal diabetes in pregnancy, pregnancy induced hypertension/pre-eclampsia/eclampsia, maternal chorioamnionitis, and multiple pregnancies with twin-to-twin transfusions. However, these variables were subject to high level of missing data in the NNRD dataset (20%) and adding them in the group of matching variables reduced substantially the resulting matching dataset for analysis.

We, therefore conducted four sensitivity analyses to explore the impact of missing data in the final matching dataset and ATET results: 1) a model using a complete case analysis from participants with

complete information related to maternal medical conditions (Model M1); 2) a model including an indicator of whether participant had missing data related to maternal medical conditions (M2); 3) a model considered all missing information about maternal medical conditions as "no medical conditions" (M3); and 4) a model with all missing information as "medical condition present" (M4). To account for the severity of the babies' condition we also repeated our main matching analysis after i) excluding babies with serious congenital anomalies as we believed these babies were more likely to be delivered in a centre with a NICU and ii) babies who stayed in either unit for less than a week.

Presence of serious congenital anomalies was defined as a diagnosis on discharge/death of any of the ICD-10 codes defined by Helenius et al.[5]

Results

Of the 20,565 babies, with complete birth (gender, gestation week, birth weight, multiple birth, mode of delivery), demographic (ethnicity, maternal age at birth, index of multiple deprivation) and mortality (i.e. primary outcome) data (Figure 1), 17,700 were exactly matched for gender, gestational age, birth weight, maternal age at birth, multiple births, ethnicity, mode of delivery and IMD (8,850 NICU babies matched with 7,654 LNU babies).

Supplementary material 3 - Figure 1: Flowchart of participants included in matching analysis

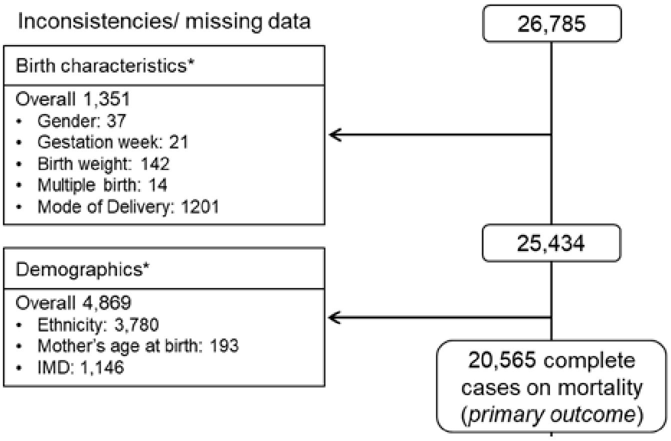

After matching, the overall difference in probability of death (ATET) for babies born in NICU remained 0.8% statistically higher (95% CI: 0.1% to 1.4%) than what it would have been if they were born in LNU (Table 1). When considering mortality by week of gestational age, there was no apparent trend of increasing or decreasing mortality and the only statistically significant difference was in babies born at 31 weeks gestational age, who had mortality 1.2% higher (95% CI: 0.4% to 2.1%) in the NICU than they would have had in the LNU. Whilst not statistically significant, the difference in mortality was highest for babies born at 28 weeks gestational age (2.1%, 95% CI: -0.1% to 4.2%, Table 1).

Supplementary material 3 - Table 1: Adjusted difference in mortality for babies between 27^{+0} and 31^{+6} weeks gestation born in NICU compared with delivery in LNU.

	NICU	LNU	Total matched cases*	ATET (95% CI)	p-value
Death before discharge					
Overall	8,850	7,654	17,700	0.8% (0.1% to 1.4%)	0.02
By week of gestation					
27^{+0} to 27^{+6}	1,079	685	2,158	0.8% (-1.9% to 3.6%)	0.5
28^{+0} to 28^{+6}	1,393	1,068	2,786	2.1% (-0.1% to 4.2%)	0.06
29^{+0} to 29^{+6}	1,563	1,370	3,126	-0.7% (-2.2% to 0.8%)	0.3
30^{+0} to 30^{+6}	2,117	1,856	4,234	0.4% (-0.6% to 1.4%)	0.5
31^{+0} to 31^{+6}	2,698	2,675	5,396	1.2% (0.4% to 2.1%)	0.004

**Total matched cases are the total of babies from the NICU and those sampled with replacement from the LNU.*

Sensitivity analyses

Ideally, the presence of maternal medical conditions would have been included in the primary matching analysis. When this variable was included, the overall difference in ATET was attenuated and became non-significant (0.5%, 95% CI: -0.3% to 1.3%), but the sample size of the total matched cases was substantially reduced because of the high levels of missing data (M1, Table 2). Incorporating maternal medical conditions fail to explain the differences between groups for babies born at 31 weeks gestational age as the statistically significant difference in ATET between groups persisted (1.7% (0.7% to 2.7%)). The remaining models M2, M3 and M4 resulted in non-statistically significant differences in overall mortality (Table 2).

The result observed in the overall sample also remained consistent after exclusion of n=275 with serious congenital anomalies (0.8%, 95% CI: 0.02% to 1.4%), though was attenuated (0.5%, 95% CI: 0.07% to 1.0%) after the exclusion of n=322 babies who stayed in the unit for less than a week.

Supplementary material 3 - Table 2: Adjusted difference in mortality for babies between 27^{+0} and 31^{+6} weeks gestation born in NICU compared with delivery in LNU, for sensitivity analyses to investigate the impact of missing data.

	NICU	LNU	Total matched cases*	ATET (95% CI)	p-value
M1: Complete cases with medical conditions	3,731	3,471	7,462	0.5% (-0.3% to 1.3%)	0.3
By week of gestation					
27^{+0} to 27^{+6}	622	429	1,244	0.9% (-2.6% to 4.5%)	0.6
28^{+0} to 28^{+6}	818	678	1,636	1.0% (-1.5% to 3.6%)	0.4
29^{+0} to 29^{+6}	935	845	1,870	-0.5% (-2.2% to 1.2%)	0.6
30^{+0} to 30^{+6}	1,328	1,137	2,656	0.6% (-0.5% to 1.7%)	0.3
31^{+0} to 31^{+6}	1,756	1,766	3,512	1.7% (0.7% to 2.7%)	0.001
M2. Include missing medical condition indicator	4,713	4,398	9,426	0.5% (-0.3% to 1.2%)	0.2
M3: All missing medical conditions as "no medical conditions"	6,298	5,681	11,979	0.4% (-0.3% to 1.1%)	0.2
M4: All missing medical conditions as "medical condition present"	6,777	6,012	12,789	0.5% (-0.2% to 1.1%)	0.2

**Total matched cases are the total of babies from the NICU and those sampled with replacement from the LNU.*

Interpretation

The results of our primary matching exercise revealed a significant difference in mortality between NICU and LNU, primarily driven by babies born between 31^{+0} and 31+6 weeks. When we incorporated information on maternal medical conditions into the matching process, we still observed a significant difference in mortality in babies born between 31^{+0} and 31^{+6} weeks but statistical differences in overall mortality disappeared. These findings indicated that our initial matching analysis lacked validity without accounting for maternal health conditions. Unfortunately, the substantial amount of missing data on maternal health conditions in our dataset resulted in a significant reduction in the sample used for the matching analysis. There were concerns hence that the results obtained were affected by using a selected group of participants with complete cases on medical conditions and the impact of unmeasured confounders. To overcome this limitation, the research team engaged in discussions with the co-Investigator's Group and the Study Steering Committee, leading to a consensus to employ an instrumental variable approach as the primary method for analysing the clinical, secondary outcomes and cost-effectiveness in the study. The instrumental variable method presented the advantage of effectively addressing both the measured confounders present in our dataset and the unmeasured confounders, such as maternal health conditions that were subject to high levels of missing data.

References

[1] Elizabeth A. Stuart. Matching Methods for Causal Inference: A Review and a Look Forward. Statistical Science 2010;25(1):1-21.

[2] Matthay EC, Hagan E, Gottlieb LM, Tan ML, Vlahov D, Adler NE, et al. Alternative causal inference methods in population health research: Evaluating trade offs and triangulating evidence. SSM - Population Health 2020;10:100526.

[3] Norris T, Seaton SE, Manktelow BN, Baker PN, Kurinczuk JJ, Field D, et al. Updated birth weight centiles for England and Wales. Archives of Disease in Childhood - Fetal and Neonatal Edition 2018;103(6):F577-F582.

[4] Abadie A, Imbens GW. Bias-Corrected Matching Estimators for Average Treatment Effects. Journal of Business & Economic Statistics 2011;29(1):1-11.

[5] Helenius K, Longford N, Lehtonen L, Modi N, Gale C. Association of early postnatal transfer and birth outside a tertiary hospital with mortality and severe brain injury in extremely preterm infants: observational cohort study with propensity score matching. BMJ 2019;367:l5678.

Supplementary material 4: The instrumental variable modelling framework - key considerations and interpretation of model outputs

Introduction

This section provides supplementary information to understand how instrumental variable (IV) models were estimated in this study. IV estimation can be difficult to conceptualise and understand at first and here we only provide a brief summary. The reader is referred to key publications elsewhere for a full understanding surrounding IV estimation in epidemiological research.[1,2]

Causal inference methods based on adjustment for confounding rely on the assumption that all variables needed to adjust for confounding are available (i.e. we have not excluded important unmeasured confounders) and have been correctly measured. When this assumption is not valid, the inferences obtained from models adjusted for confounders are likely to be biased. IV estimation provides an alternative approach to make causal inference that does not rely on the assumption that all measured confounders of interest have been measured and captured.

In OPTI-PREM, the aim was to estimate whether neonatal care received in a NICU resulted in better outcomes (e.g. overall mortality) compared with neonatal care received in a LNU for babies born at 27-31 weeks. In the causal diagram supplementary material 4 – Figure 1, this treatment effect is represented by β_{tx}, and if all measured confounders of interest are included in a regression model (indicated by arrows from the measured confounders to the unit designation or the outcome), this estimated coefficient will be an unbiased estimate of the treatment effect of receiving care in NICU versus LNU. We showed in supplementary material 3 that limited information about maternal health conditions, an important factor that can affect the outcome for a baby, was available in our dataset. Missing maternal health conditions can be considered an unmeasured confounder that can affect outcomes but also whether the baby is delivered in a NICU or LNU setting. When important unmeasured confounders (indicated by arrows from the unmeasured confounders to unit designation or the outcome in supplementary material 4 – Figure 1) are correlated with the unit designation (NICU/LNU), then the coefficient β_{tx} in a regression model is biased. One of way to think about this is that such coefficient does not only capture the treatment effect due to care received in a unit designation but also residual confounding. Making inferences based on this parameter is problematic if residual confounder is important.

Supplementary material 4 – Figure 1

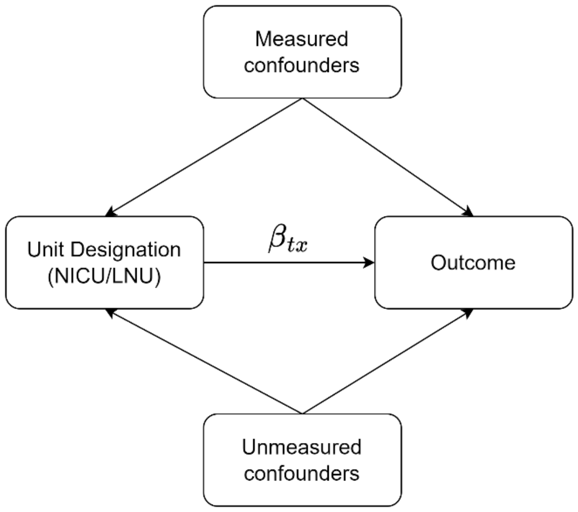

An alternative way of identifying the average causal effect of receiving care in a unit designation on outcomes without the need to have available all measured confounders of interest is the IV approach described in the causal diagram supplementary material 4 – Figure 2. In the IV approach, an instrument (that need to meet certain conditions as described in the next section) is identified such as it only affects the outcome through unit designation. Therefore, the instrument cannot directly influence outcomes as indicated with the dash line with a red cross from the IV box to the outcome box in the figure. In addition, the instrument needs to be correlated with unit designation but cannot be correlated with unmeasured confounders (indicated by the dash line with a red cross in the figure from the unmeasured confounder box to the IV box). Intuitively, the instrument aims to correct for all the influence of the unmeasured confounders in the neonatal care received as it is not related to any of them. In other words, the instrument needs to be correlated with the unit designation but cannot be correlated with the unmeasured confounders so when an IV model is estimated, the resulting estimate of β_{tx} does not capture any potential residual confounding.

Supplementary material 4 – Figure 2

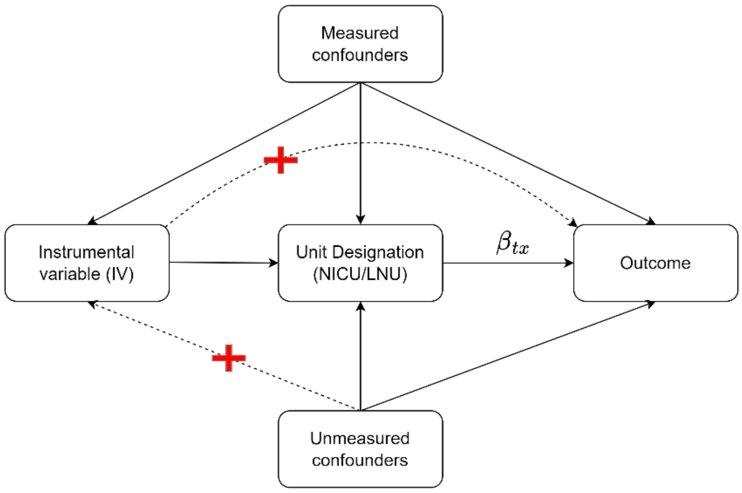

Choice of instrumental variable

In this study, we operationalised excess travel time (in minutes) between mother's residence to closer NICU minus mother's residence to closer LNU as the instrument in our IV model. Positive differential time indicated mother lived closer to a LNU whereas negative values indicated mother lived closer to a NICU. Supplementary material 5 provides information about how this instrument was estimated in the OPTI-PREM study. For this variable to be a valid instrument, it needs to meet the three below conditions.

- **Condition 1:** the instrument needs to be associated with the type of unitat birth (unit designation). Excess travel time is likely to be correlated with unit designation (NICU/LNU). If a woman lives closer to a LNU, it is likely that their baby will be delivered in that setting. This was corroborated cross-tabulating unit designation with excess travel time and calculating Pearson correlation coefficient as presented in Supplementary material 4 – Table 1. The table shows that women delivering in a LNU lived closer to a LNU as indicated by a positive median excess travel time of 14.28 minutes. Similarly, women living closer to a NICU lived closer to that setting as indicated by the negative median excess travel time of -7.69 minutes. In our analysis unit designation was defined as a binary variable with value 0 for LNU and 1 for NICU. Therefore, the statistically significant negative Pearson correlation coefficient of -0.54 indicates women living closer to a NICU had significantly negative excess travel times as expected.

Supplementary material 4 – Table 1: Descriptive summary of excess travel time (in minutes) by unit designation

	**LNU**	**NICU**
**Median**	14.28	-7.69
**Mean**	16.75	-10.56
**Stand. Dev.**	16.59	24.29
**Pearson Correlation**	-0.54 (p<0.001)	

The strength of excess travel time was also evaluated using a simple logistic regression where the dependent variable was unit designation (binary NICU/LNU) and the independent variables included excess travel time as continuous and all the measured confounders used for adjusting. The latter included gestational age, sex, birthweight z-score, multiplicity, mode of delivery, maternal ethnicity, maternal age and index of multiple deprivation. The results of this model resulted in a statistical significant coefficient associated with excess travel time of an odds ratio of 0.911 with 95% confidence interval 0.907 to 0.914. This indicated that as excess travel time increases (LNU closer than NICU), the odds of living closer to a NICU decreases by around 9%.

- **Condition 2:** the instrument does not have a direct effect on the outcome (e.g. mortality or secondary outcomes). Proximity to a unit designation is assumed to affect outcomes only through the care received in a particular setting. Simply living near a NICU does guarantee a good outcome because the baby has to be delivered in a NICU to benefit.

- **Condition 3:** the instrument is independent of unmeasured confounders (although can be related to measured confounders). Excess travel time is not directly related to any unmeasured confounders such as maternal health conditions. Women generally do not choose to live close to a hospital with a NICU in case they ever have a preterm birth. Of course, it is not possible to observe whether unmeasured confounders are evenly distributed between groups using an IV, but this is possible with measured confounders. To this end we assessed whether the instrument distributed measured confounders evenly using the median of the excess travel time distribution across the whole cohort as a cut-off value. An absolute standardised difference of ≥0.10 was used to identify covariates imbalanced between groups.[3] Table 2 in the main text presented neonatal and maternal characteristics by median excess travel time to a neonatal intensive care unit (NICU).

IV Model Estimation

The most common method to estimate IV models is the two-stage least squares (2SLS) regression.[4] The approach involves first evaluating the association between unit designation and excess travel time (instrument) and obtain fitted values from that model. This stage ensures the isolation of the variation in unit designation that can be explained by the instrument. Fitted values of unit designation from the first stage are then used in a second model that assess the outcome of interest (e.g. unit designation on mortality). This second model included besides the fitted values of unit designation, all the measured confounders used to adjust (gestational age, sex, birthweight z-score, multiplicity, mode of delivery, maternal ethnicity, maternal age and index of multiple deprivation).

All the outcomes of interest including mortality and secondary outcomes were defined as binary outcomes (0 event not present; 1 event present). In addition, unit designation was also defined as a binary outcome. Therefore, a categorical limited-dependent variable was needed to estimate our parameters of interest for the first and second stage IV modelling. In this study we implemented a seemingly unrelated probit fitted using maximum-likelihood two-equation probit models.[5,6] This model was estimated in Stata MP18 using the command biprobit. Similar to the standard probit model, the coefficients from a *biprobit* model have primarily a qualitative interpretation. To estimate the average causal effect, expressed in this study as mean probability in each unit designation and mean probability differences with associated confidence intervals between units, we used the *margins* postestimation command in Stata. This estimation approach was utilised for the primary mortality and each of the secondary outcomes.

References

[1] Hernan, M.A. and Robins, J.M. Causal Inference: What if. 2020, Boca Raton: Chapman & Hall/CRC.

[2] Greenland, S. An introduction to instrumental variables for epidemiologists. Int J Epidemiol, 2000. 29(6): 1102.

[3] Austin, P.C. Balance diagnostics for comparing the distribution of baseline covariates between treatment groups in propensity-score matched samples. Stat Med, 2009. 28(25): 3083-107.

[4] Greene, W.M. Econometric Analysis. 7th ed. 2012, New Jersey: Prentice Hall.

[5] Angrist, J.D. Estimation of Limited Dependent Variable Models With Dummy Endogenous Regressors. Journal of Business & Economic Statistics, 2001. 19(1): 2-28.

[6] Freedman, D.A. and Sekhon, J.S. Endogeneity in Probit Response Models. Political Analysis, 2010. 18(2): 138-150.

Supplementary material 5: Estimation of differential travel time (excess travel time) to a neonatal intensive care unit (NICU)

Our instrumental variable was defined as the difference in time (in minutes) between mother's residence to closer NICU minus mother's residence to closer LNU. Therefore, positive differential time indicated mother lived closer to a LNU whereas negative values indicated mother lived closer to a NICU.
The calculation of travel times was conducted by the team at the Neonatal Data Analysis Unit (NDAU) at Imperial College as they were the data controller with permission to use sensitive information including maternal postcode data in our study. The team employed Open Source Routing Machine (OSRM) and OpenStreetMap [https://www.openstreetmap.org] to find the optimal route by car using the Stata command osrmtime [1]. In a nutshell, the command calculates the travel time and distance from a point of origin to a point of destination using OSRM data. OSRM is a high-performance open-source C++ routing engine that indicates the shortest routes on public roads and runs with open-source maps from OpenStreetMap. Complete details of the OSRM programme and how to access it can be found at https://github.com/christophrust/osrmtime. We used the latest map of England at the time of the conduct of the study that can be found at https://download.geofabrik.de/europe/great-britain/england-latest.osm.pbf. Maternal postcodes were converted into georeferenced latitude and longitude before they were imported into the command using Open Postcode Geo (https://www.getthedata.com/open-postcode-geo). Descriptive statistics of travel times by maternal rural urban classification used by the Department for Environment, Food & Rural Affairs in England (https://www.gov.uk/government/statistics/2011-rural-urban-classification) are presented below indicating that 89.5% of babies included in OPTI-PREM were born in urban locations.

Descriptive statistics of travel time to NICU/LNU and excess travel time of babies included in OPTI-PREM

Maternal Rural-Urban Classification	Freq.	Percent	Median time to NICU (mins)	Median time to LNU (mins)	Median excess travel time (mins)	Min excess travel time (mins)	Max excess travel time (mins)
Urban conurbation	8,950	47.5%	14.57	12.65	2.67	-130.36	35.03
Urban city and town	7,907	42.0%	27.00	24.08	4.46	-122.60	74.11
Rural town and fringe	1,179	6.3%	34.25	26.21	10.26	-122.98	68.66
Rural village and dispersed	811	4.3%	38.18	27.42	12.90	-100.00	71.34
Total	18,847	100%	19.97	16.48	3.91	-130.36	74.11

Reference:

[1] Huber S, Rust C. Calculate travel time and distance with OpenStreetMap data using the Open Source Routing Machine (OSRM). Stata Journal. 2016;16(2):416-23.

Supplementary material 6: Comparison of cohort excluded due to missing data with those included in the final analysis

Characteristic	Included cohort (n=18,847)	Missing cohort (n=7,438)
**Place of birth, n (%)**		
Unit co-located with LNU	8,468 (44.9)	3,368 (45.3)
Unit co-located with NICU	10,379 (55.1)	4,070 (54.7)
**Gestational age, n (%)**		
27 weeks	2,284 (12.1)	912 (12.3)
28 weeks	3,031 (16.1)	1,128 (15.2)
29 weeks	3,412 (18.1)	1,339 (18.0)
30 weeks	4,379 (23.2)	1,789 (24.1)
31 weeks	5,741 (30.5)	2,249 (30.2)
Missing/inconsistent	-	21 (0.3)
**Died in neonatal care, n (%)**	574 (3.1)	210 (2.8)
**Died in one year, n (%)**	695 (3.7)	264 (3.6)

Supplementary material 7: Additional information about transfers within 72 hours of life

The National Neonatal Research Database (NNRD) was organised using different data tables depending on the nature of the observation included in the extract (e.g. episode or daily records). Transfers in this study were identified from the NNRD daily records that contained information about the daily interventions received by babies. A code that identified the hospital providing care for a particular baby daily was available. We compared the hospital code assigned to the place of birth of the first daily record with any subsequent daily records for the duration of neonatal stay. A transfer was identified when the initial hospital code differed from the hospital code of a subsequent daily record. When a transfer was identified, we also extracted information about the type of transfer carried out (e.g. LNU to NICU, NICU to LNU and so on…). In this study, we only conducted an analysis of the first transfer identified for each baby between providers.

A total of 6,016 babies were transferred from their original place of birth at some point during their neonatal stay (3,738 NICU and 2,278 LNU).

1,545 babies were transferred up to 72 hours from birth (928 out of LNU and 617 out of NICU).

A breakdown of transfers by gestational age and place of birth is provided in the next table for transfers by 72 hours of birth (Supplementary material 7 – Table 1)

Supplementary material 7 – Table 1

	NICU (n = 10,379)			LNU (n = 8,468)		
**Gestational age**	**Total births**	**Number (% of total births) transferred at any time**	**Number (% of all transfers) transferred in ~72 hours**	**Total births**	**Number (% of total births) transferred at any time**	**Number (% of all transfers) transferred in ~72 hours**
27	1,507	747 (49.6)	82 (11.0)	777	389 (50.1)	228 (58.6)
28	1,773	717 (40.4)	91 (12.7)	1,258	491 (39.0)	231 (47.0)
29	1,885	689 (36.6)	107 (15.5)	1,527	438 (28.7)	167 (38.1)
30	2,348	790 (33.6)	132 (16.7)	2,031	449 (22.1)	139 (31.0)
31	2,866	795 (27.7)	205 (25.8)	2,875	511 (17.8)	163 (31.9)
Total	10,379	3,738 (36.0)	617 (16.5)	8,468	2,278 (26.9)	928 (40.7)

A breakdown of transfers by gestational age and volume of admissions is provided in Supplementary material 7 – Table 2.

Supplementary material 7 – Table 2

	High-volume (n = 4,597)			Low-volume (n = 14,184)		
**Gestational age**	**Total births**	**Number (% of total births) transferred at any time**	**Number (% of all transfers) transferred in ~72 hours**	**Total births**	**Number (% of total births) transferred at any time**	**Number (% of all transfers) transferred in ~72 hours**
27	671	359 (53.5)	45 (12.5)	1,604	768 (47.9)	259 (33.7)
28	787	359 (45.6)	39 (10.9)	2,234	839 (37.6)	277 (33.0)
29	837	332 (39.7)	46 (13.9)	2,556	776 (30.3)	217 (28.0)
30	1,067	402 (37.7)	63 (15.7)	3,299	824 (25.0)	200 (24.3)
31	1,235	380 (30.8)	90 (23.7)	4,491	911 (20.3)	267 (29.3)
Total	4,597	1,832 (39.9)	283 (15.4)	14,184	4,118 (29.0)	1,220 (29.6)

The direction of transfers by 72 hours of birth are presented in Supplementary material 7– Table 3:

Supplementary material 7 – Table 3

		All transfers		Up to 72 hours	
**From**	**To**	**n**	**Percentage**	**n**	**Percentage**
NICU	LNU	1,990	33.08	306	19.81
LNU	NICU	1,620	26.93	834	53.98
NICU	NICU	971	16.14	290	18.77
NICU	SCU	777	12.92	21	1.36
LNU	LNU	423	7.03	90	5.83
LNU	SCU	235	3.91	4	0.26
**Total**		**6,016**	100	**1,545**	100

Supplementary material 7 – Table 3: Direction of transfers between units. NICU = Neonatal Intensive Care, LNU = Local Neonatal Unit, SCU = Special Care Unit

The direction of transfers in the first 72 hours of life by gestational age is presented in Supplementary material 7 - Figure 1. Of 1,545 transfers in the first 72 hours, 928 (60.1%) were transferred out of LNU; of these, 834 (89.9%) were transfers from LNU-to-NICU.

There was a total of 2,284 births at 27 weeks gestation, of whom 310 (13.5%) were transferred by the first 72 hours of birth. 228 of these (73.5%) were transfers out of LNU, of which 219 (96.1%) were uplifts from non-tertiary (LNU) to tertiary (NICU) care (LNU-to-NICU).

Capacity transfers are usually determined by either caseload of babies requiring intensive care/high dependency care, or inadequate nursing staffing to cope with care of the babies within the unit. These are determined by the neonatal team and generally a risk assessment is made on a case-by-case basis. For anticipated births, the ideal is to transfer mothers with baby in-utero, however this is dependent on the capacity of co-located maternity services to accept an in-utero transfer, and whether or not it is safe to transfer the mother, or whether there is time to transfer between presentation of a mother at a maternity service and the preterm birth. Capacity transfers usually include transfers from NICU to LNU, between LNU and LNU. Transfers between NICU to NICU, are usually for specialist care but could also represent capacity transfers. It was not possible to determine the latter within our data set.

Supplementary material 7 – Figure 1

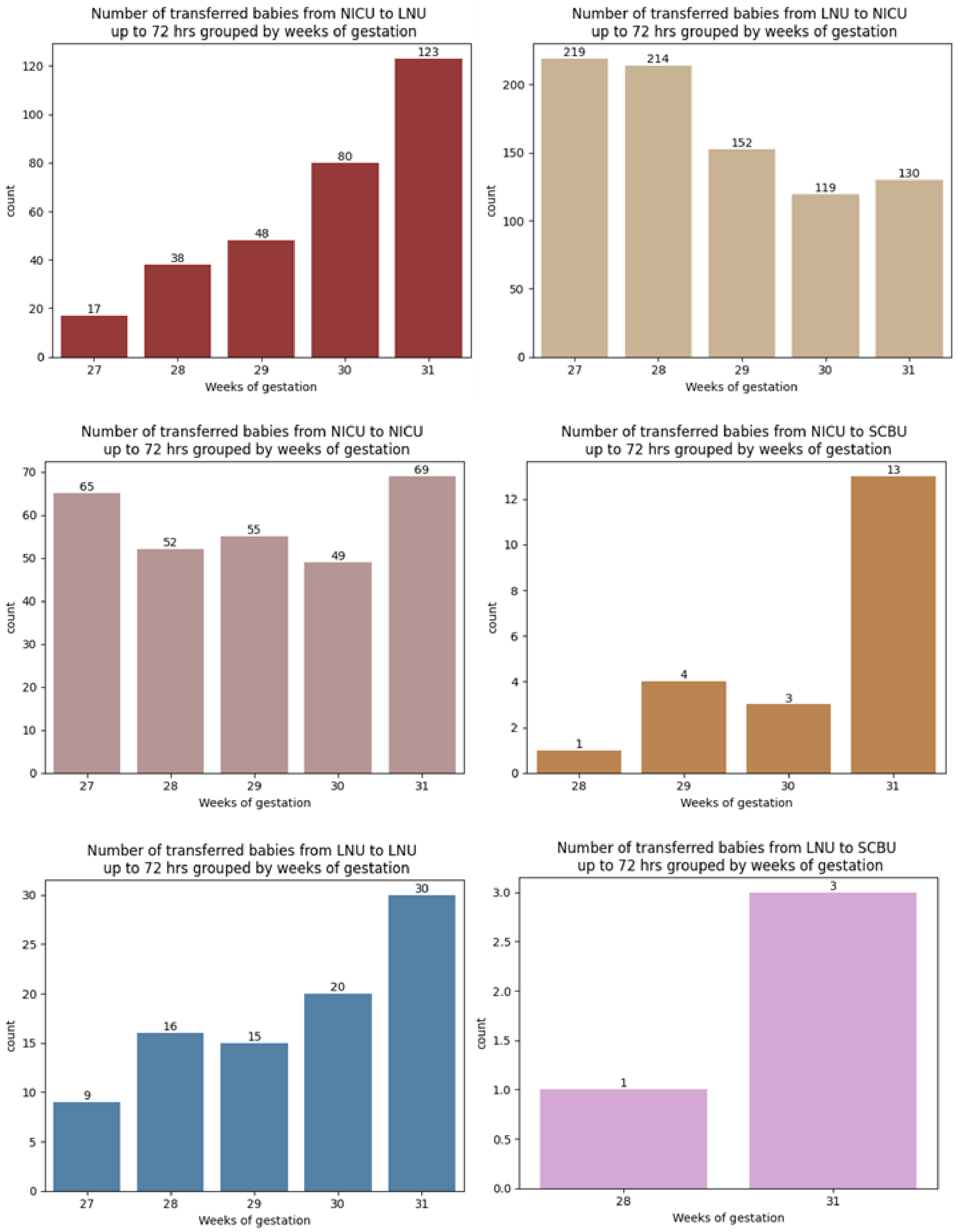

Transfers at each gestational age and cross-tabulation with transfers up to 72 hours in those babies with serious brain injury (SBI) by place of birth is presented in Supplementary material 7 – Table 4. There were a total of 2284 births at 27 weeks gestation, of whom 310 (13.5%) were transferred by the first 72 hours of birth. There were 228 births at 27 weeks gestation at LNU of whom 219 (96.1%) were uplifts to NICU. In this cohort of babies transferred out of LNU to NICU by 72 hours of birth, there were 38 SBI (i.e 17.4% SBI for transfers of births at 27 weeks gestation from LNU to NICU by 72 hours of birth).

Supplementary material 7 – Table 4

**NICU (n = 10,379)**				
**Gestational age**	**Total births**	**Number (% of total births) transferred in ~72 hours**	**Number of SBI (% of total births)**	**Number of SBI (% of those who were transferred in ~72)**
27	1,507	82 (5.4)	123 (8.2)	7 (8.5)
28	1,773	91 (5.1)	86 (4.9)	8 (8.8)
29	1,885	107 (5.7)	71 (3.8)	4 (3.7)
30	2,348	132 (5.6)	58 (2.5)	3 (2.2)
31	2,866	205 (7.2)	47 (1.6)	3 (1.5)
**Total**	**10,379**	**617 (5.9)**	**385 (3.7)**	**25 (4.1)**
**LNU (n = 8,468)**				
27	777	228 (29.3)	83 (10.7)	38 (16.7)
28	1,258	231 (18.4)	94 (7.4)	33 (14.3)
29	1,527	167 (10.9)	75 (4.9)	24 (14.4)
30	2,031	139 (6.8)	56 (2.8)	10 (7.2)
31	2,875	163 (5.7)	42 (1.5)	10 (6.1)
**Total**	**8,468**	**928 (11.0)**	**350 (4.1)**	**115 (12.4)**

Supplementary materials 8: Association between place of birth (high volume admissions versus low volume admissions) and overall and gestational age-specific mortality risk whilst in neonatal care and at one year using instrumental variable model (n=18,781)

	High volume mean percentage	SE	Low volume mean percentage	SE	Adjusted mean difference	99% confidence interval	p-value for difference
**Died in NNU**							
Overall	2.2%	0.5%	3.5%	0.5%	-1.5%	-4.4% to 1.5%	0.2
27 weeks	3.8%	1.6%	11.4%	4.2%	-9.0%	-29.5% to 11.4%	0.3
28 weeks	3.0%	1.1%	7.8%	1.9%	-5.8%	-16.8% to 5.2%	0.1
29 weeks	1.3%	0.7%	4.5%	4.5%	-4.1%	-19.3% to 11.1%	0.5
30 weeks	0.8%	0.3%	2.0%	0.6%	-1.4%	-4.8% to 2.0%	0.3
31 weeks	2.0%	0.8%	1.2%	0.2%	0.6%	-1.2% to 2.5%	0.4
**Died in One Year**							
Overall	2.5%	0.5%	4.5%	0.5%	-2.3%	-5.6% to 1.0%	0.07
27 weeks	4.9%	2.3%	11.8%	4.1%	-7.9%	-29.4% to 13.5%	0.3
28 weeks	3.1%	1.0%	9.4%	2.1%	-7.9%	-19.5% to 3.7%	0.08
29 weeks	1.6%	0.6%	6.2%	3.1%	-6.2%	-21.6% to 9.3%	0.3
30 weeks	1.1%	0.5%	2.6%	0.8%	-1.8%	-6.3% to 2.7%	0.3
31 weeks	1.9%	0.7%	1.9%	0.3%	0.0%	-2.4% to 2.4%	0.99

* Adjusted for gestational age (when analysing the overall cohort); sex; birthweight z-score; multiplicity; mode of delivery; maternal ethnicity; maternal age and index of multiple deprivation; SE: standard error

Supplementary material 9: Distribution of actual cases for the secondary outcomes in preterm babies born at 27-31 weeks gestation in maternity services co-located with NICU and LNU, and admitted into neonatal care

Secondary Outcome	LNU	NICU	Total cases/ Denominator	Explanation of denominator
Any morbidity (ROP or oxygen dependency or SBI or NEC) or died	1293	2114	3407/18847	n/a
ROP	106	191	297/17930	Excludes 574 babies who died and 343 missing ROP record
Oxygen dependency	611	1208	1819/18273	Excludes 574 babies who died
NEC	205	285	490/18847	Includes babies who died
Serious brain injury	350	385	735/18847	Includes babies who died
Breast milk at discharge	4726	5494	10220/18273	Excludes 574 babies who died

Supplementary material 10: Association between place of birth (high volume admissions versus low volume admissions) of risk of serious brain injury whilst in neonatal care under different scenarios using instrumental variable model

	High volume mean (SE) percentage	Low volume mean (SE) percentage	Adjusted mean difference* (99% CI)	p-value for difference
Overall cohort (n=18,781)	1.6% (0.3%)	6.4% (1.0%)	-6.5% (-12.1% to -0.9%)	0.003
Excluding babies born at 27 weeks gestation (n=16,506)	1.4% (0.3%)	4.8% (0.8%)	-4.5% (-9.6% to 0.5%)	0.02
Excluding post-natal transfers (n=17,278)	1.8% (0.4%)	5.2% (0.9%)	-4.2% (-9.3% to 0.8%)	0.03
Babies born at 27-weeks (n=2,275)	2.8% (0.4%)	24.2% (6.7%)	-28.9% (-54.2% to -3.5%)	0.003

* Adjusted for gestational age (when analysing the overall cohort); sex; birthweight z-score; multiplicity; mode of delivery; maternal ethnicity; maternal age and index of multiple deprivation; SE: standard error; CI: confidence interval
